# Insights on dying, dementia and death certificates

**DOI:** 10.1186/s13690-018-0263-7

**Published:** 2018-03-12

**Authors:** Sofie Vandormael, Alexander Meirschaert, Jan Steyaert, Jan De Lepeleire

**Affiliations:** 1 KU Leuven, Leuven, Belgium; 2 Expertisecentrum Dementie and University of Antwerp, Antwerp, Belgium; 3 Academisch Centrum Huisartsgeneeskunde (ACHG) KU Leuven, Kapucijnenvoer 33, blok J, PB 7001, 3000 Leuven, Belgium

**Keywords:** Mortality statistics, Death certificates, Dementia

## Abstract

For our master thesis in medicine, we aimed to determine how many deaths were caused by and with dementia in 2014 and we compared our results with figures from abroad. The mortality rates of 2014 in Flanders were used to determine the amount of deaths related to dementia. These figures are collected by Vlaams Agentschap Zorg & Gezondheid (VAZG) and coded per ICD-10 classification. Of all deaths in Flanders in 2014, 6.60% were caused by dementia and 4.29% were caused by another condition, while also suffering from dementia. Data from abroad are ambiguous. While working on our thesis about “death & dementia”, we questioned the reliability of mortality statistics. Possible explanations could be; the complexity of completing death certificates correctly and the challenges involved in properly constructing a chain of causes of death. The accuracy of mortality data can be improved by training and redrafting death certificates.

## Background

Mortality statistics are an important source of information, often used for planning health care budgets. Concerns regarding the accuracy of these statistics are growing. The article by Perera et al., published in Age & Ageing in May 2016, aimed to investigate the frequency with which clinically diagnosed dementia was recorded on death certificates [1]. Their retrospective cohort study investigated the death certificates of people with a clinical diagnosis of dementia (in patients aged 65 or older), who died between 2006 and 2013. In this group, dementia was recorded on 53.6% of those death certificates. They concluded with the following statement: “Mortality data still considerably underestimate the population burden of dementia. Potential biases affecting recording of dementia need to be considered when interpreting mortality data.”

Inspired by this study, we set out to investigate the situation in Flanders, Belgium. Our objectives were to describe how mortality statistics are collected in Flanders and to compare the percentage of deaths related to dementia in Flanders to similar statistics in other countries. During this study we questioned the reliability of mortality statistics. Therefore, we extended our scope: how reliable are figures based on death certificates and how can the accuracy of mortality statistics be improved?

## Comparison to Flemish situation

### Collecting data about causes of death in Flanders

We focused on the mortality statistics of 2014 in Flanders, one out of three regions in Belgium. The data for the other regions, Brussels and Wallonia, are collected and coded in a slightly different way and are out of scope for this study. The figures from Flanders, Brussels and Wallonia are ultimately combined by Statistics Belgium (*Algemene Directie Statistiek* or ADS). The most recent figures are those of Flanders in 2014 and thus these were used for this study [2].

**Fig. 1:**
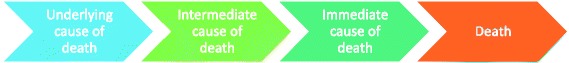
Chain of causes of death

Mortality statistics are sourced from the information stated on death certificates. In Flanders, these documents are called “model III C” (for deceased age one year or over). These forms should be filled in by a physician. The physician needs to document the nature and cause of death. Multiple causes of death can be noted and should be organised in a “chain of causes of death”. This chain starts with the underlying cause of death (the condition responsible for the death), followed by intermediated causes of death (caused by the underlying cause of death) and ultimately ends with the immediate cause of death (the ultimate disease or injury resulting in death) (Fig. 1). Besides this chain, there is the option to mention associated causes of death. These conditions did not directly lead to the passing of the individual, however, they did contribute to the frailty of the patient.

The death certificates are collected by the Flemish Agency for Care and Health (*Vlaams Agentschap Zorg en Gezondheid* or VAZG) and then coded using the international classification of disease (ICD-10) [3]. Coding can be done manually by coders or semi-automatically by IRIS (a coding system that converts the physician’s literal words -through a conversion table- to ICD-10 codes). After statistical processing the results are published online (www.zorg-en-gezondheid.be/cijfers).

**Fig. 2:**
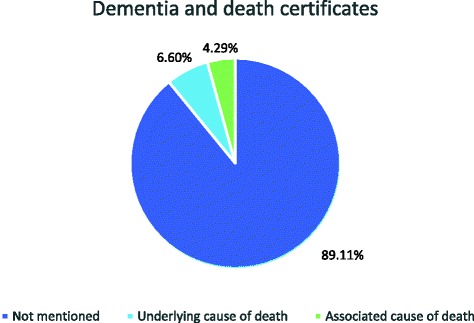
Dementia and death certificates in Flandres (2014)

For this commentary, we examined the deaths caused by dementia (i.e. dementia as an underlying cause of death) and the deaths with dementia (i.e. dementia as an associated cause of death) in Flanders in 2014. After ICD-10, the following codes were used for dementia: F00.X, F02.X, F03.X, F05.1, F10.7 and G30.X. On most death certificates (89.11%) dementia is not mentioned, on 6.60% of the forms it was mentioned as an underlying cause of death and on 4.29% it was mentioned as an associated cause of death (Fig. 2).

### Comparing deaths caused by dementia in Flanders with similar foreign studies

We calculated the deaths caused by dementia (i.e. dementia as an underlying cause of death) in Flanders in 2014 and compared them with results from similar foreign studies (Table 1). Norwegian data from 1969 and 2010 illustrate that throughout the years dementia is more frequently mentioned on death certificates. This increase is also described in the article by Perera et al. [1]. Furthermore, we notice the following trend in all countries: deaths caused by dementia seem to be more common among the elderly and women. This can be explained by the increased prevalence of dementia with aging and the fact that women have a longer life expectancy and a slightly higher risk for dementia than men.

**Table 1:** Comparing dying of dementia in Flanders with similar foreign studies

Source Article	Country Region	Year	< 65 male	< 65 female	> 65 male	> 65 female	> 65 M + F	All ages Male	All ages Female	All ages M + F	60–79 male	60–79 female	> 79 male	> 79 female
VAZG [2]	Flanders (BE)	2014	1.00%	1.42%	5.45%	9.63%	7.63%	4.62%	8.69%	6.60%	2.48%	4.04%	7.23%	11.37%
CBS [4]	The Netherlands	2014								4.92%				
Désesquelles et al. [5]	France	2008					6.96%							
Désesquelles et al. [5]	Italy	2008					4.62%							
Gillum et al. [6]	US	2005–2006				15.90%								
Hjellvik et al. [7]	Norway	1969			3.00%	4.00%								
Hjellvik et al. [7]	Norway	2010			7.00%	15.00%								
Kmietowicz [8]	UK & Wales	2013						6.20%	12.20%					
Roberto et al. [9]	Brazil	2000–2009									7.70%	8.40%	14%	15.5%

The exact percentages, however, are rather different and these geographic differences are already mentioned in some papers. For example: for the same gender and age group (both genders, age above 65) we see 4.62% of deaths due to dementia in Italy (2008) and 15.9% in the US (2005–2006). Is there a real difference in prevalence of (deaths caused by) dementia? Or, maybe the method used to collect and code this data different in these countries? Or, perhaps this is a comparison of death caused by dementia, deaths with dementia or a combination of both?

### Comparing deaths with dementia in Flanders with similar foreign studies

We started by searching through scientific literature for frequent causes of death in people with dementia. Most frequently mentioned were the respiratory infections (especially pneumonia), falling, cardiovascular diseases, Parkinson’s disease, dehydration, cachexia. We calculated the amount of deaths with dementia as an associated cause of death, caused by pneumonia, falling and Parkinson’s disease (Table 2): 0.04% of the deaths in Flanders in 2014 were caused by pneumonia and associated with dementia, 0.34% caused by Parkinson’s disease and associated with dementia and 0.08% caused by falling associated with dementia.

**Table 2:** Dementia as an associated cause of death in Flanders (2014)

Associated cause of death	Underlying cause of death	Frequency	Percentage
Dementia	Pneumonia	25	0.04%
Dementia	Parkinson’s disease	200	0.34%
Dementia	Falling	47	0.08%

These percentages only account for 0.46% of all deaths and are nowhere near the 4.29% of deaths associated with dementia. The discrepancy between our results and those described in similar literature is striking. Are the most frequently described underlying causes of death not the most frequent in Flanders? Perhaps this difference is due to a different way of collecting and coding causes of death? We suspect that the complex chain of causes of death and multiple definitions (underlying, intermediate, immediate and associated causes of death) used in the Flemish forms can be challenging. This could be an interesting research topic for the future.

The reliability of mortality statistics is questionable. Despite this critique and doubt, not much effort has been made to improve the quality of mortality statistics.

### Suggestions to improve the accuracy of mortality statistics

It can be concluded that caution is advised when interpreting mortality data. We should strive to improve mortality statistics, as these are often used for the health care policy. More care should go into filling in death certificates correctly. It is known that training can improve the accuracy of mortality data. These training programmes could be incorporated into medical students’ curriculum and be presented as an extra course for practicing physicians. The complex Flemish/Belgian model III C could be improved by simplifying it: allowing the physician to mention all diseases and injuries contributing to the cause of death without having to rank them into the chain of causes of death, but with the possibility to allocate a measure of importance to each cause. For the interpretation of the statistics, we could also use other databases, for example, the morbidity registration database Intego (Flanders), which contains information regarding the prevalence of different conditions. This might help us understand which conditions are over- or underreported as causes of death.

## Conclusion

We do agree with Perera’s findings [1]: mortality data may underestimate the population burden of dementia and caution is advised when interpreting mortality data. Accurate mortality statistics are of the utmost importance and we should strive to improve them. Providing training and adjusting death certificates could prove to be worthwhile.

## Abbreviations

ADS: *Algemene Directie Statistiek*
ICD-10: International classification of disease
VAZG: *Vlaams Agentschap Zorg & Gezondheid*
